# The Lipoprotein Transport System in the Pathogenesis of Multiple Myeloma: Advances and Challenges

**DOI:** 10.3389/fonc.2021.638288

**Published:** 2021-03-26

**Authors:** Vasileios Lazaris, Aikaterini Hatziri, Argiris Symeonidis, Kyriakos E. Kypreos

**Affiliations:** ^1^Pharmacology Laboratory, Department of Medicine, School of Health Sciences, University of Patras, Patras, Greece; ^2^Hematology Clinic, Department of Medicine, School of Health Sciences, University of Patras, Patras, Greece; ^3^Department of Life Sciences, School of Sciences, European University Cyprus, Nicosia, Cyprus

**Keywords:** multiple myeloma, pathophysiology, lipoproteins, signaling, lipids, bone marrow, HDL

## Abstract

Multiple myeloma (MM) is an incurable neoplastic hematologic disorder characterized by malignant plasma cells, mainly in the bone marrow. MM is associated with multiple factors, such as lipid metabolism, obesity, and age-associated disease development. Although, the precise pathogenetic mechanisms remain unknown, abnormal lipid and lipoprotein levels have been reported in patients with MM. Interestingly, patients with higher APOA1 levels, the major apolipoprotein of high density lipoprotein (HDL), have better overall survival. The limited existing studies regarding serum lipoproteins in MM are inconclusive, and often contradictory. Nevertheless, it appears that deregulation of the lipoprotein transport system may facilitate the development of the disease. Here, we provide a critical review of the literature on the role of lipids and lipoproteins in MM pathophysiology. We also propose novel mechanisms, linking the development and progression of MM to the metabolism of blood lipoproteins. We anticipate that proteomic and lipidomic analyses of serum lipoproteins along with analyses of their functionality may improve our understanding and shed light on novel mechanistic aspects of MM pathophysiology.

## Introduction—Overview of Multiple Myeloma

Multiple myeloma (MM), the second most common hematological malignancy, comprising 13–15% of all blood cancers, is characterized by the proliferation of atypical plasma cells, leading to impairment of normal hematopoiesis, bone and renal disease, immunosuppression, and various organ-specific damage. MM results from the accumulation of mutations and chromosomal aberrations during B-cell differentiation, and its clinical phenotype ranges from the completely asymptomatic monoclonal gammopathy of undetermined significance (MGUS) and the smoldering or asymptomatic myeloma (SM), to the fully symptomatic MM (1), an incurable disease with a 5 year survival probability of about 50–55% (2). Developments in the disease management during the last 20 years, including the incorporation of various targeting agents into the classical chemotherapeutic regimens have changed the prognosis and improved the lifespan of patients with MM (3). The main categories of new anti-myeloma agents include proteasome inhibitors, immunomodulatory drugs (IMiDs), and monoclonal antibodies, while several other therapies, such as BCL-2 inhibitors, modulators of nuclear export, other small molecules, and CAR-T cells are currently in clinical trials of late phase or have already received approval.

Pathogenetically, although the initial molecular trigger remains unclear, dysregulation of the expression pattern of certain cyclins, and particularly of the cyclin D superfamily of proteins, appears critical and consistently occurring (4, 5). The nature of genetic defects identifies distinct molecular subgroups and differentiates the MM into two subtypes: hyperdiploid, characterized by complex karyotype, with multiple trisomies or gain of other genetic materials, and non-hyperdipoid. The latter group exhibits chromosomal translocations, involving the immunoglobulin heavy chain (IGH) region, located in the long arm of chromosome 14, such as t(11;14) (q13; q32) and t(6;14) (p21; q32), creating the proximity of the cyclin D1 (CCND1) and cyclin D3 (CCND3) genes, respectively, to the IGH locus, and directly leading to the overexpression of cyclin genes. Notably, the overexpression of CCDN1 is associated with enhanced adhesion of plasma cells to stromal cells, chemotaxis, and drug resistance (4). The MMSET and musculoaponeurotic fibrosarcoma oncogene homolog (MAF) genes (c-Maf and MafB), coding for histone methyltransferase, and leucine zipper-containing transcription factors, respectively, are also commonly found dysregulated through translocation of their chromosomal loci at 4p16, 16q23, and 20q12, to the 14q32 region, leading to the overexpression of cyclin D2 gene (CCND2), and identify a high-risk disease (6). As in many other cancers, deletion of chromosome 17p and the subsequent loss of the tumor suppressor gene, TP53, usually occurring in later phases of the disease, confers dismal prognosis. The t(4;14) or t(14;16) translocation, along with del17p or with 1q21 chromosomal region amplification (7) have been incorporated in the Revised International Staging System (R-ISS) (8) that predicts survival for patients with newly diagnosed MM. Moreover, data from Next Generation Sequencing (NGS) have revealed secondary mutation in RAS and Nuclear factor kappa-light-chain-enhancer of activated B cell (NF-κB pathways, as well as mutations of the TP53, ATM, MYC, RB1, and ATR genes (6).

A key discovery of great importance was the interaction of the marrow microenvironment with neoplastic plasma cells (9). It is now well-known that clinical course of MM is not only determined by the neoplastic potential of the clone, but also by the tumor (usually bone marrow) microenvironment. Bone marrow microenvironment consists, among others, of stromal cells, osteoclasts, osteoblasts, fibroblasts, endothelial cells, and bone marrow adipocytes, and each of these cell types contributes to the pathogenesis and progression of the disease. Through this complex landscape, new evidence suggests that bone marrow adipocytes might have a role in disease progression. While obesity is a well-established risk factor for patients with MM, dyslipidemia appears to be an emerging prognostic factor for disease development and outcome, implying that dysregulation of the lipoprotein transport system could play a pivotal role even in disease manifestation. Furthermore, other factors, such as lipid metabolism, obesity, and age play a role in the disease pathophysiology, reflecting the complexity of MM. This review aims to provide a critical evaluation of the current literature on the role of lipids and lipoproteins in the development of MM and to identify the potential novel research avenues toward this direction that need further investigation.

## Myeloma Cell Microenvironment and the Emerging Role of Bone Marrow Adipocytes

Multiple myeloma cells grow primarily in the bone marrow. The unique milieu of bone marrow supports the growth of MM cells, through excretions of cytokine and exosome and cell-to-cell interactions. The stromal cells of the bone marrow play a key role in these processes. These fibroblast-like cells interact with MM cells, leading to the secretion of anti-apoptotic and myeloma growth-promoting cytokines, such as interleukin-6 (Il-6), insulin-like growth factor-1(IGF-1), and stromal cell-derived factor-1 (SDF-1) (9).

Interleukin-6 is a pleiotropic cytokine with a protagonistic role in inflammation, immune response, and hematopoiesis. Apart from stromal cells, the malignant plasma cells, myeloid, and endothelial cells also produce Il-6 (10). In MM, Il-6 contributes to the proliferation, migration, and drug resistance of the malignant cells. Moreover, Il-6 promotes angiogenesis and the growth of tumor, through the induction of vascular endothelial growth factor (VEGF) gene expression. Osteoclastogenesis is also promoted by Il-6, through macrophage activation and induction of osteoclast maturation, resulting in the dysregulation of bone metabolism and extensive bone loss (11). The myeloid cells, induced by Il-6, support the growth of MM cells by producing the proliferative factor, a proliferation-inducing ligand (APRIL). APRIL belongs to the tumor necrosis factor (TNF) ligand superfamily and binds to the B-cell maturation antigen (BCMA) receptor. BCMA belonging to the TNF receptor superfamily (also known as TNFRSF17 and CD269), is expressed preferentially on mature B-lymphocytes and preserves the long-lived plasma cells (12). The binding of APRIL to BCMA promotes the cell growth of MM and induces immunosuppression (12) mediated by the production of transforming growth factor beta 1 (TGFβ1) and other cytokines (13, 14).

Stromal cell-derived factor-1 (SDF-1) or CXCL12 is another important chemokine, excreted by the stromal cells of bone marrow, endothelial cells, and MM cells. Its receptor, CXCR4, is expressed by various malignant cells, including plasma cells. Their interaction is important for the homing of the malignant cells and the progression of myeloma. Inhibition of SDF-1 binding to the CXCR4, with the CXCR4 inhibitor plerixafor, results in the mobilization of plasma cells and loss of stromal cell support. Also, resistant myeloma clones exhibit high expression of CXCR4 (15, 16).

Given the complexity of tumor microenvironment, malignant plasma cells also interact with other BM cells, through exosomes. These are secreted by membrane vesicles that carry messages in the form of proteins, such as micro RNAs (miRNAs) and cytokines. Exosomes take part in physiological functions mediating cell-to-cell communication. Interestingly, it is shown that the content of exosomes of bone-marrow-derived mesenchymal stromal cells (BMSC)—is different between patients with myeloma and healthy donors (17). Myeloma-derived exosomes are found to promote angiogenesis, osteolysis, plasma cell proliferation, and drug resistance (17–19). Furthermore, compounds that alter the production or the uptake of exosomes are under investigation in preclinical and clinical trials (20).

It has recently been shown that bone marrow adipocytes (BMAs) interact with MM cells. Derived from non-hematopoietic BMSCs, BMAs function as energy depot and as an endocrine organ with characteristics of both, white and brown adipose tissues (21). Patients with MM tend to have increased pre-adipocytes and significantly larger mature adipocytes in their bone marrow (22). Interestingly, BMAs support tumor growth and protect malignant cells from chemotherapy-related apoptosis (23), through the induction of autophagic proteins in plasma cells (24). The supportive role of BMAs has also been demonstrated in acute myeloid leukemia. The myeloid blasts alter the metabolism of BMAs, turning them to fatty acid donors (25). On the contrary, BMAs inhibit the proliferation of T-acute lymphoblastic leukemia T-ALL blasts in an *in vivo* animal model (26).

**Figure 1:**
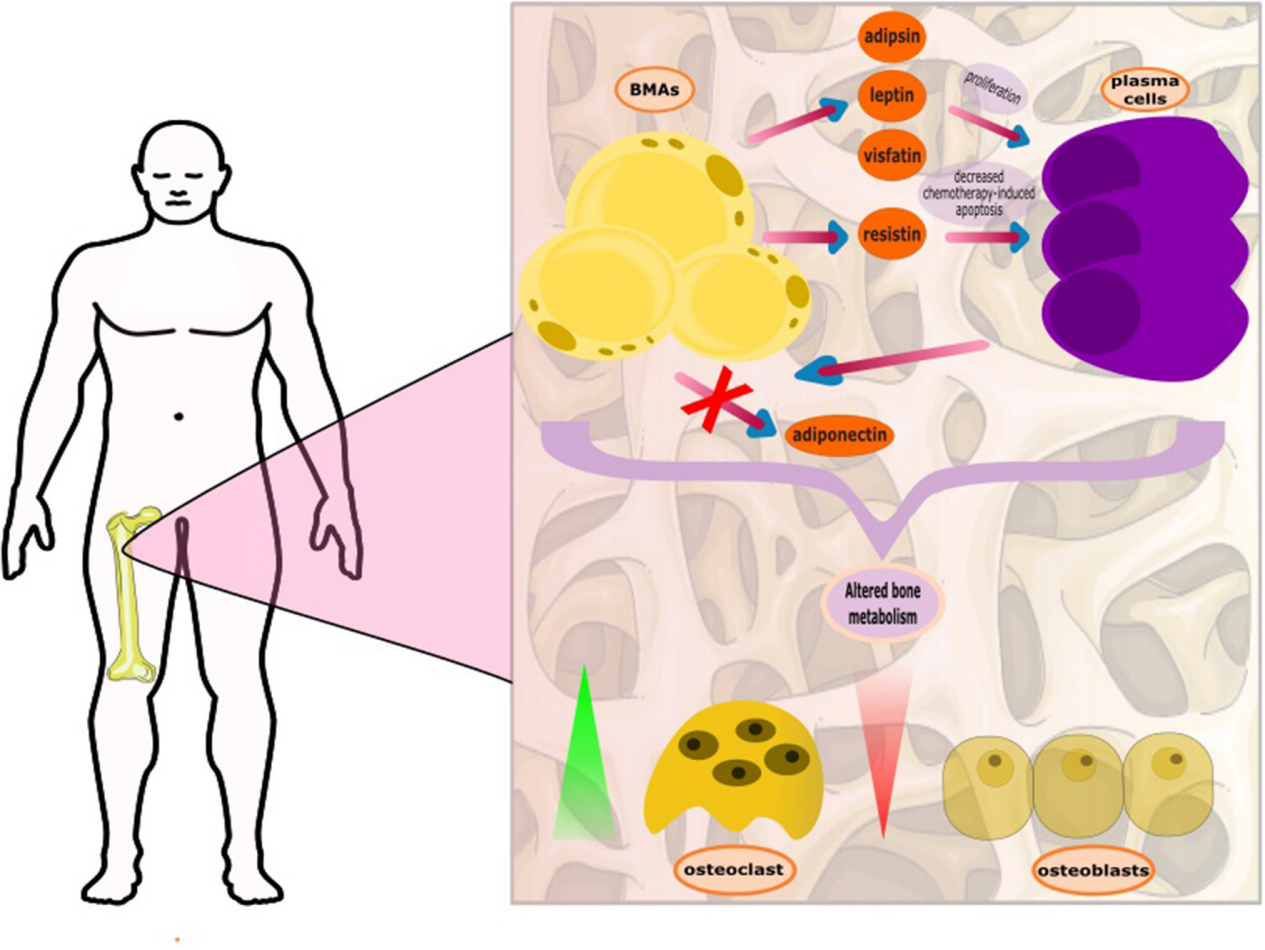
The complex interplay between bone marrow adipocytes (BMAs) and plasma cells. The BMAs, through secretion of adipokines, support the growth and proliferation of the plasma cells. The plasma cells, with unknown mediator, appear to deter adiponectin production. Adiponectin is considered to block myelomatogenesis by reducing intracellular levels of nuclear factor kappa-light-chain-enhancer of activated B cells (NF-kB) and increasing the levels of anti-inflammatory cytokines. It has recently been found that plasma cells reprogram BMAs resulting in persistent bone disease.

Moreover, the adipocytes may also have a role in bone remodeling. It is recently shown that MM cells can reprogram adipocytes, which prevent bone lesion reversal after disease remission. These reprogramed adipocytes express less peroxisome proliferator-activated receptor γ (PPARγ), due to methylation of its promoter (27). Another aspect of the complex interplay between the neoplastic plasma cells and BMAs is the downregulation of the expression of adiponectin by plasma cells. Adiponectin, an adipokine involved in fatty acid metabolism, has antitumor effects and is produced by BMAs and other adipocytes (28). As part of its activities, adiponectin suppresses the production of Il-6, and therefore, its reduction may indirectly contribute to the progression of myeloma cells. Aging, obesity, dyslipidemia, and metabolic syndrome appear to correlate with the growth of both, MM and BMA; yet the exact role of BMAs is still under investigation in the MM setting. It is also known that impairment in the high density lipoprotein (HDL) metabolic pathway, that results in the formation of lower levels of HDL-cholesterol (HDL-C) and dysfunctional HDL particles in serum, is associated with increased deposition of BMAs, a finding further indicating an important role of dyslipidemia and reduced HDL-C, in particular, in the development and progression of MM (29, 30) (Figure 1).

It is possible that additional factors related to the condition of tissue microenvironment, such as local pH, levels of glucose and fatty acids, and the availability of electrolytes and minerals, may also impact the progression of disease. Despite the substantial improvement in the clinical diagnostics and treatment strategies, real-time monitoring of tumor microenvironment is not currently feasible. The enormous progress in the miniaturization of electronics is expected to lead to ultra-miniaturized biodegradability in blood sensors (also known as electroceuticals), for this purpose.

## Multiple Myeloma and the Lipoprotein Transport System

Lipoproteins are responsible for the transport of lipids in circulation, and are involved in three different, but interconnected metabolic pathways: (1) the chylomicron pathway, which is important for the absorption and distribution of dietary lipids, (2) the very low density lipoprotein (VLDL)/intermediate density lipoprotein (IDL)/low density lipoprotein (LDL) pathway, which is crucial for the delivery of endogenously synthesized lipids from the liver to the peripheral tissues, and (3) the HDL pathway, which plays a pivotal role for the redistribution of peripheral cholesterol and other lipids among various tissues, including the tissues in liver. Many different proteins, including apolipoproteins, enzymes, lipid transfer proteins, and lipoprotein receptors participate in these pathways and contribute to the overall lipid homeostasis (31).

Chylomicrons are synthesized within the enterocytes, following the loading of lipids onto Apolipoprotein B48 (Apo B-48) molecules by the action of the intestinal microsomal triglyceride transfer protein (MTTP). Following their synthesis, chylomicrons are secreted through the lymphatic circulation into the blood stream, where they are converted to chylomicron remnants by lipoprotein lipase (LPL), and acquire apolipoproteinE (ApoE), which mediates their clearance by the members of the low density lipoprotein receptor (LDLR) superfamily. During VLDL assembly, hepatic lipids are transferred onto Apolipoprotein B100 (Apo B100) with the action of hepatic MTTP, leading to the formation of nascent VLDL particles, which are then secreted directly into the systemic circulation. Like chylomicrons, VLDL triglycerides (TGs) are hydrolyzed by the action of plasma LPL, and are initially converted to IDL and then to LDL particles, which are removed from the circulation by the members of the LDL receptor superfamily. Chylomicron remnants, VLDL, and LDL are cleared from circulation, primarily through members of the LDL receptor superfamily (LDLR and LRP1), although the relative contribution of LRP1 in the overall process remains under discussion (32). Heparan sulfate proteoglycans (HSPG) have also been suggested to play a role in this process, possibly by attracting circulating lipoproteins on cell membranes (33, 34) and presenting them to the LDL receptors (35) (expressed ubiquitously), LRP1 (expressed in the liver, the brain, and the placenta), VLDLR (expressed in the heart, the muscle, and the adipose tissue) and scavenger receptors (expressed in the liver, steroidogenic tissues, and macrophages) (31). HSPG, mainly syndecan-1, has been shown to act as an independent receptor for TG-rich lipoprotein remnants in the liver (36). Additionally, LPL is bound to HSPG, and this interaction is thought to be mediated through ApoE, which further strengthens the stability of the lipoprotein-LPL group (37, 38). A novel role of HSPG is the response to major stress factors of the tumor microenvironment, such as hypoxia, through increased recruitment of HDL, LDL, and VLDL particles (39).

Syndecan-1 is found on the surface of MM cells and has a key role in the interaction of MM cells with the bone marrow microenvironment (40–44). Myeloma tumors are characterized by high levels of expression of cell surface syndecan-1, with its HS chains playing the most important role for the growth of MM cell and survival within the BM microenvironment (40, 42, 45). It has also been found that syndecan-1 is the co-receptor for APRIL, which is an important factor in the BM microenvironment, favoring MM cell survival (46). It is noteworthy that the soluble form of syndecan-1 promotes the growth of myeloma *in vivo* (47). Moreover, suppression of syndecan-1 resulted in the inhibition of the growth of MM cells and increased rate of apoptosis (43, 44, 48). The conversion of HSPG—mainly defined by the degradation of the HS chains *via* enzymes, such as sulfatases and heparanase—within the tumor microenvironment is generally thought to influence signaling events and has a significant impact on the development of cancer. Thus, these enzymes can be used as therapeutic targets for the treatment of cancer, in general, and MM, in particular (49). Overexpression of heparanase in the bone marrow of patients with MM was associated with a shorter event-free survival, possibly because heparanase induces osteoclastogenesis and bone loss, leading to changes in signaling in the BM microenvironment (49). Recently, the heparanase inhibitor, roneparstat was tested in Phase I clinical trial, on patients with MM (50).

**Figure 2:**
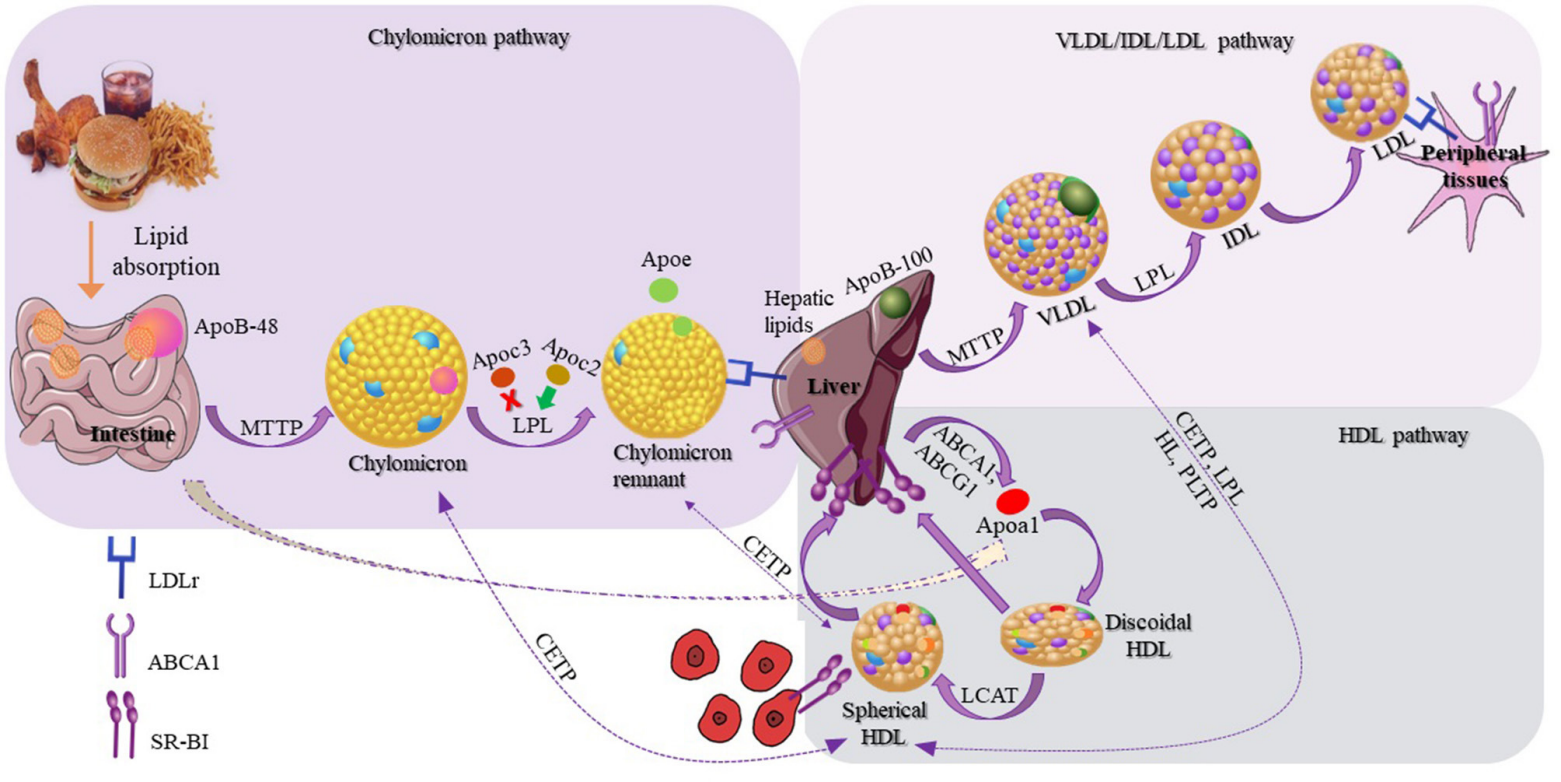
Schematic representation of lipoproteins and their three different metabolic pathways. The schematic representation of the chylomicron pathway (in the upper-left purple frame) indicates their biosynthesis from the intestine and their clearance by the members of the low density lipoprotein (LDL) receptors. The schematic representation of the very low density lipoprotein (VLDL)/intermediate density lipoprotein (IDL)/LDL pathway (in the upper-right lighter purple frame) indicates the VLDL synthesis by the liver and finally the clearance of LDL by the LDL receptors. The schematic representation of the HDL pathway (in the lower-right blue frame) indicates the steps for the discoidal synthesis of high density lipoprotein (HDL), the mature spherical HDL, and finally their removal from the circulation by interacting with SR-BI receptor. Additional steps are also shown (dashed arrows) in which HDL exchanges lipids with chylomicrons, chylomicron remnants, and VLDL.

In contrast to chylomicrons and VLDL, biogenesis of HDL occurs exclusively in systemic circulation (51) with the participation of apolipoproteins, lipid transporters, such as ATP-binding cassette A1 (ABCA1) and G1 (ABCG1), and the plasma enzyme, such as lecithin-cholesterol acyltransferase (LCAT). HDL metabolism also involves additional steps, in which plasma enzyme cholesteryl ester transfer protein (CETP) mediates the exchange of cholesteryl ester (CE), present in HDL, for TGs present in triglyceride-rich lipoproteins (TRLs) (chylomicrons, chylomicron remnants, and VLDL). This enzymatic exchange is a critical crossing point for all three lipoprotein pathways, where TGs from TRLs enter the HDL pathway, and CE from HDL enter the chylomicron and VLDL pathways. This exchange results in the modulation of size and geometry of HDL, leading to particles more suitable for binding to the HDL receptor, SR-BI (Figure 2).

Although serum lipoproteins are usually referred in the literature as single species, it is now well-accepted that each lipoprotein category comprises numerous subclasses, with variable protein and lipid contents, distinct physicochemical characteristics, and metabolic actions (52–56). For example, seven different subclasses of LDL species have been detected, using ultracentrifugation and electrophoretic techniques (52–54) and 48 different proteins are associated with LDL particles (57). Similarly, many different subclasses of HDL particles have been identified (51, 58–63). Importantly, structural differences among HDL subclasses correspond to functional differences (64).

Genetic alterations affecting plasma lipid and lipoprotein homeostasis (increased levels of chylomicrons, VLDL, and LDL or reduced levels of HDL) are associated with the development of dyslipidemia, a condition known to affect cancer development and progression. Various types of neoplastic cells elaborate distinct mechanisms for lipid uptake and utilization (65); some of which may trigger minor or no effect on the serum lipid and lipoprotein profiles, whereas others may lead to substantial changes (66). Several recent studies have indicated that dyslipidemia may also be associated with many hematological malignances and solid tumors (67–70). Therefore, the critical role of the lipid and lipoprotein transport system demonstrated in many types of cancer (69) also extends to the pathogenesis of MM.

Preliminary data indicate that changes in lipoprotein metabolism may trigger specific cellular and molecular events, that in turn could influence bone marrow microenvironment and adipocyte formation (71). Although the number of studies in patients with MM remains very limited and the quality of clinical evidence is low, abnormal lipid and lipoprotein profiles have clearly been associated with MM (Table 1).

**Table 1:** Clinical studies evaluating the changes in plasma HDL-Cholesterol (HDL-C), LDL-Cholesterol (LDL-C), Triglycerides (TGs), and Total Cholesterol (TC) levels in patients with multiple myeloma (MM).

**Number of subjects**	**Lipids tested**	**Main results**	**References**
32 patients with MM vs. 110 healthy people	HDL-C, LDL-C, TGs, and TC in three different time points	HDL-C ↓ in patients, in the active period of the disease LDL-C, TC no difference TGs ↑ in patients, at the time of diagnosis and in the active period	(72)
102 patients with MM vs. 71 healthy people	HDL-C, LDL-C, VLDL-C, TGs, and TC in different stages of the disease	HDL-C ↓ in patients in Stage III LDL-C ↓ in patients in Stage II TC↓ in patients in Stages II, III VLDL-C, TGs no difference	(73)
307 patients with MM vs. no control group	HDL, LDL, TGs, TC, APOA1, and APOB in different stages of the disease	HDL ↑ in Stage I LDL, TC, APOA1, APOB ↓ in Stage III TGs no difference When APOA1 ↑ led to longer overall survival	(74)
20 patients with MM vs. 30 healthy people	Chylomicron lipolysis	No difference	(75)
*In vitro* study with three different myeloma cell lines	Incubation with or without cholesterol in the serum	LDL-C improved cell viability and prevented cell apoptosis	(76)

*↑ and ↓ denote increased and decreased levels, respectively*.

One such clinical study investigated how plasma HDL-C, LDL-C, TGs, and TC levels may change in patients with MM at different stages of the disease, namely at the time of diagnosis, in the active period, and during disease remission, and compared them to those of healthy individuals. The data suggested no stage-dependent changes in LDL-C and total cholesterol levels between the groups, although HDL-C levels were lower in patients with MM, only during the active period of the disease, compared to healthy subjects. Triglyceride levels were higher at the time of diagnosis and during the active disease period in patients with MM, compared to healthy controls. Nevertheless, during disease remission, the serum lipid levels were similar between patients with MM and controls, showing a correlation between active disease periods and disturbances in the plasma lipoprotein cholesterol and in the triglyceride levels (72).

Yavasoglu et al. (73) assessed serum lipid levels in 102 patients with MM, at different types and stages of the disease, and observed a disparity among the three International Staging System (ISS) stages of MM. LDL-C and HDL-C levels were significantly lower in patients at Stages II and III, respectively, compared to healthy individuals, while total cholesterol levels were significantly lower in patients at Stages II and III, compared to healthy subjects. No differences were observed in VLDL-cholesterol (VLDL-C) and triglyceride levels between the two groups (73).

In 2019, a study enrolling 307 patients with MM, evaluated the prognostic significance of plasma, such as Apolipoprotein A1 (APOA1), Apolipoprotein B (APOB), cholesterol, triglycerides, HDL, and LDL for the different stages of the disease, according to the ISS. The results indicated that only patients with MM, expressing higher levels of APOA1 exhibited longer overall survival (determined as the time of diagnosis to the most recent follow-up or the time of death), progression-free survival (determined as the time of diagnosis to the time of the most recent follow-up or the time of disease progression as per the criteria of International Myeloma Working Group) and cause specific survival (determined as the time of diagnosis to the most recent follow-up or the time of death caused by myeloma). Other parameters of lipid metabolism, such as APOB levels, total cholesterol, and LDL levels were lower in the late ISS stage, but showed no statistically significant correlation with either overall survival or progression-free survival. Lastly, no differences were observed in serum triglyceride levels between the different ISS stages (74).

Chylomicron metabolism in MM has been investigated only in one study including 20 subjects with MM, compared to 30 healthy subjects. According to this study, chylomicron lipolysis was not affected in patients with MM, albeit a reduced rate of remnant clearance was observed (75). In another study, the role of LDL-C on myeloma cell viability was evaluated *in vitro*, using three different human myeloma cell lines that were cultured with or without cholesterol in the culture medium. It was found that exogenous LDL-C improved cell viability and prevented apoptosis, indicating that LDL-C is a potent anti-apoptotic agent for the cells of human myeloma (76).

Another potential link between dyslipidemia and hematological cancer is the rising evidence for the fact that inhibitors of 3-hydroxy-3-methylglutaryl coenzyme A reductase (HMG-CoA reductase)—commonly used in the treatment of hyperlipidemia—were found to have anti-myeloma activity, both, *in vivo* (77–80) and *in vitro* (81–85), probably by inducing apoptosis of myeloma cells.

Despite the emerging role of Lp(a) in the pathophysiology of inflammatory diseases, such as atherosclerosis, it should be noted that to date, no data exist on the role of Lp(a) in the development and progression of MM. Similarly, although hypertriglyceridemia has been reported in patients with hematological malignancies, such as acute promyelocytic leukemia (86), only few cases of patients with MM who developed hypertriglyceridemia have been reported, and its role in disease development remains unclear (87, 88).

It should be noted that selected bioactive lipids present on lipoproteins may also contribute to the development of MM cells. Alterations of normal PL plasma and sphingolipid metabolism, observed in the various lysosomal storage disorders (LSD), particularly in Gaucher disease, in which these alterations have been more extensively studied, are considered to play a pivotal role in the increased incidence of both hematological malignancies and solid tumors, observed among patients with this disease (89). Patients with Gaucher disease, in particular, are prone to manifesting MGUS and MM about 10–50 times more frequently than the general population (90) and this high relative risk has undergone intensive investigation. In this disease biosynthesis and development of abnormal glucosphingolipids, such as lysophosphatidylcholine (LPC) and glucosylsphingosineis, patients demonstrate effort to metabolize the accumulated undigested disease substrate, glucosylceramide. However, these abnormal glucosphingolipids appear to trigger the immune system for the production of monoclonal immunoglobulins directed against them (91), but these can also support cancer cells through different mechanisms (92). What is impressive is that abnormal glucosphingolipids have more recently been implicated in the origin of MM even outside the setting of Gaucher disease (93). Most importantly, the oncogenic potential of glucosphingolipids appears not to be restricted in MM but to be extended to melanoma and other cancers (92, 94). Finally, and most interestingly, substrate debulking in Gaucher disease, achieved with the administration of Enzyme Replacement Therapy (ERT), appears to be capable to attenuate the generation of monoclonal immunoglobulins, implying a potential attenuation of cancer signaling in patients, in whom the amount of glucosphingolipids is reduced (95). These findings provide strong evidence about the oncogenic potential of some glucosphingolipids for cancer, in general, and particularly for MM.

## Multiple Myeloma and Obesity: the Role of Lipoproteins

In recent years, it has become apparent that morbid obesity constitutes a major risk factor for cancer (96), with 13 different types, including MM, being etiologically associated with the obese phenotype (USA CDC 2017) (97). Meta-analyses claim that increased body mass index (BMI) is a risk factor for MM (98). Other studies suggested that increased BMI during midlife is a factor for developing MM in individuals with MGUS (99). Adipose tissue from obese individuals support MM cell line proliferation and adhesion (100). According to a preclinical study in mice, diet-induced obesity promoted a myeloma-like condition (101). Insulin resistance, alterations in IGF-1 signaling pathway, and biosynthesis of sex hormones, low grade chronic inflammation, and oxidative stress are thought to be among the biological factors linking these two pathologies (102).

Increasing evidence supports that the lipid and lipoprotein transport systems play a major role in the development of morbid obesity, being responsible for the management of exogenously acquired (dietary) and endogenously synthesized lipids. Among other factors, APOA1, the most abundant protein component of HDL and ApoE, a protein component of HDL/ LDL/VLDL and the functional ligand of LDLR in the clearance of TRLs from the circulation, are also major contributors to diet-induced obesity (103–110). Therefore, it is conceivable to hypothesize that APOA1 and ApoE influence the processes associated with the development and progression of MM.

## Multiple Myeloma and Cell-Membrane Fluidity: the Role of Lipoproteins

A possible mechanism that could mediate, at least in part, the effects of the lipid and lipoprotein transport system in the development of MM need further investigation, and may involve the composition and function of the cell membrane. The cell membrane and its protein components are the primary recipients of those extracellular stimulants of cell division and proliferation. A key physical property of cell membrane is fluidity, which is also fundamental for its biological activity (111). It is well-established that changes in the cholesterol content of cell membrane may impact membrane fluidity (112), which in turn may affect the structure, antigenicity, and responsiveness of cell-surface proteins, such as ion-channels and G-coupled receptor proteins to natural stimuli (113). This in turn may affect intracellular signaling and biological responses, such as in the case of insulin secretion by pancreatic β-islets (114). Dynamic microdynamics of the membrane consisting of arranged proteins and lipids—enriched with cholesterol, sphingomyelins, and glycosphingolipids, floating within the membrane bilayer or packed together—have been described in almost all mammalian cell membranes and characterized as lipid rafts (115). Fluidity is affected by the type and local concentration of lipids, being present in the membrane bilayer, such as cholesterol and phospholipids with polyunsaturated fatty acid (PUFA) chains (116). In general, the extent of saturation of fatty acids affects their packing in the membrane bilayer and subsequently in membrane fluidity (117).

In addition to phospholipids, cholesterol also regulates membrane fluidity, by altering the packing of lipid bilayer, at a fashion dependent on the melting temperature (Tm) of the bilayer. At temperature lower than Tm, it increases the fluidity of membranes, while at temperature higher than Tm, it reduces the membrane fluidity (118).

Serum lipoproteins play important role in the remodeling of cell membrane lipid composition and fluidity by either delivering lipids to peripheral tissues, such as in the case of chylomicron remnants, VLDL, and LDL, or by exchanging cholesterol and phospholipids among tissues, such as in the case of HDL. The membrane fluidity is also affected by environmental conditions—temperature and osmotic stress—as well as by chemical agents (119, 120).

In general, there is a strong association between the degree of membrane fluidity and functional properties of both, normal and neoplastic cells (121, 122), which impacts their ability to move (normal) or metastasize (neoplastic) (121, 123). Changes in membrane fluidity bring about changes in the microenvironment of cell surface receptors and may affect, to a substantial extent, their responsiveness to extracellular stimuli (124). It is therefore possible that under conditions of altered plasma cell membrane fluidity, extracellular cell-division signals, which would normally be ignored, are effectively transduced inside the cell, triggering cell division and proliferation. However, until now, there is no clear consensus, on how changes in membrane fluidity (more or less fluid membrane) may affect the activity of cancer cells. The existing data concerning the differences in membrane fluidity status of cancer cells appear to be highly dependent on the type of cancer. Although increased membrane rigidity—increased arrangement of lipids and decreased fluidity—has been reported in hepatoma cancer cell lines, compared to normal membranes (125), increased membrane fluidity has been reported in human MT3 breast cancer cell line (126), as well as in lung cancer (127), neural tumors (128), lymphomas (129), and leukemia (130). Moreover, by using the resistant hepatocyte rat model for liver carcinogenesis, it has been suggested that the status of the cell membrane fluidity depends on the stage of cancer, since at different pathologic stages, cell membrane fluidity was found to vary substantially (131). Further confirmation of this suggestion was emerged from another study on 75 patients with lung cancer, classified at different disease stages, in which it was reported that patients with more fluid plasma membranes had worse prognosis, than those with less fluid ones (132). Taken together, these data support the crucial role of cell membrane fluidity in various cancer types, proposing the fluidity variable as a potential factor for the growth and metastasis of cancer.

Moreover, several studies have shown that multidrug resistance (MDR)—one of the major limitations in cancer chemotherapy (133)—correlates well with membrane fluidity (134, 135), and with the phospholipid, cholesterol, and unsaturated lipid content of cell membranes (121, 136).

## Concluding Remarks and Future Perspectives

**Figure 3:**
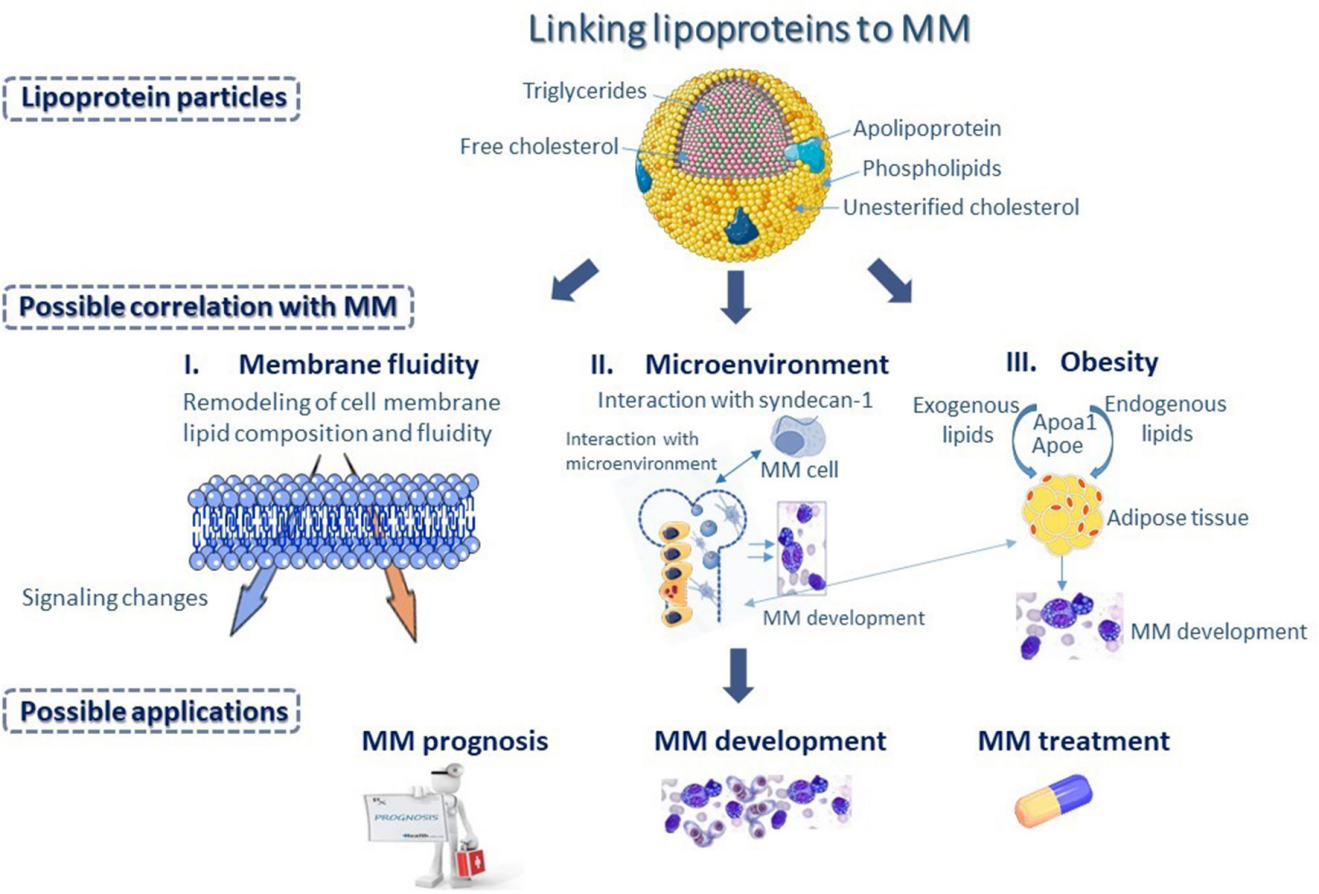
Differences in apolipoprotein and lipid composition of the various lipoprotein classes could represent a potential biomarker, linking lipoproteins to multiple myeloma (MM). Changes in lipoprotein metabolism could influence membrane fluidity of MM cells (I), as serum lipoproteins play important role in the remodeling of lipid composition and fluidity of cell membrane, and subsequently could bring about changes in intracellular signaling, affecting functional properties of MM cells. Changes in lipoprotein metabolism could also bring about changes in the bone marrow (BM) microenvironment (II) possibly through interaction with syndecan-1, found on the surface of MM cells that has a key role in the interaction of MM cells with the BM microenvironment. Additionally, changes in lipoprotein metabolism affect adipocyte formation and play a major role in the progression of morbid obesity (III) supporting MM development. Possibly, Apolipoprotein A1 (APOA1) and Apolipoprotein E (ApoE), which are major contributors to diet-induced obesity may influence processes associated with the development and progression of MM.

The pathogenesis of MM is multifactorial and depends on many different parameters (Figure 3). To this date, the lipid content of lipoproteins is the most common biomarker, linking lipoproteins to MM in small clinical studies. However, lipids are structural components of lipoproteins, expressing only their relative content in circulation. Recent advances in Lipidology indicate that in addition to lipoprotein amount in the blood, their functionality is of paramount importance for their biological activities (64). A typical paradigm is HDL, whose functionality appears to be very crucial for its involvement in numerous biological pathways and processes, while HDL-C alone provides inadequate information for the role of HDL in health and diseases. It is therefore, not surprising that those clinical studies, focusing on the role of HDL-C levels in MM are often contradictory or inconclusive. Certainly, having a lot of functional HDL is better than having little and dysfunctional, but frequently having less and functional is better than more and dysfunctional. Despite the very important observation that higher plasma APOA1 levels were found to have better overall survival, a literature search returned no studies aiming at correlating MM survival to the functionality of HDL particle.

Lipoproteins have numerous properties that ensure proper lipid homeostasis and cell membrane functionality in various cells. Moreover, they influence processes affecting cellular microenvironment, such as inflammation, oxidative stress, and regulation of sterol-responsive transcription factor activity, which may be quite important for the transformation of plasma cells into malignant cells. It is possible that differences in apolipoprotein and lipid composition of the various lipoprotein classes, among patients, may correlate with the severity and outcome of disease. Proteomic and lipidomic analyses coupled with functional analysis of serum lipoproteins, isolated from patients at different stages of the disease, may help clarify novel mechanistic aspects of MM development. It would be interesting for instance, if plasma APOA1 levels were indeed a dependable marker of overall patient survival. Currently, serum lipoproteins are not incorporated into clinical decision for the management of patients with MM. However, understanding the role of lipoproteins in the pathophysiology of MM may provide physicians an extra tool to decide for the appropriate course of action.

## Author Contributions

All authors listed have made a substantial, direct and intellectual contribution to the work, and approved it for publication.

## Conflict of Interest

The authors declare that the research was conducted in the absence of any commercial or financial relationships that could be construed as a potential conflict of interest.

## Abbreviations

ABCA1: ATP-binding cassette A1
ABCG1: ATP-binding cassette G1
ApoB-48: Apolipoprotein B-48
ApoB-100: Apolipoprotein B-100
Apoc2: Apolipoprotein C2
Apoc3: Apolipoprotein C3
ApoE: Apolipoprotein E
APRIL: A proliferation-inducing ligand
ATM gene: Ataxia-telangiectasia mutated gene
ATR gene: Ataxia telangiectasia and Rad3-related protein gene
BCL-2: B-cell lymphoma 2 gene
BCMA: B-cell maturation antigen
BMAs: Bone marrow adipocytes
BMI: Body mass index
BMSC: Bone marrow-derived mesenchymal cells
CAR-T cells: Chimeric antigen receptor T cells
CCND1: Cyclin D1 gene
CCND2: Cyclin D2 gene
CCND3: Cyclin D3 gene
CE: Cholesteryl ester
CETP: Cholesteryl ester transfer protein
CXCR4: C-X-C motif chemokine receptor 4
EL: Endothelial lipase
HDL: High density lipoprotein
HDL-C: High density lipoprotein-cholesterol
HL: Hepatic lipase
IDL: Intermediate density lipoprotein
IGH: Immunoglobulin heavy chain
IGF-1: Insulin-like growth factor-1
Il-6: Interleukin-6
IMiDs: Immunomodulatory dugs
ISS: International staging system
LCAT: Lecithin-cholesterol acyltransferase
LDL: Low density lipoprotein
LDL-C: Low density lipoprotein-cholesterol
LDLR: Low density lipoprotein receptor
Lp(a): Lipoprotein (a)
LPL: Lipoprotein lipase
MAF: Musculoaponeurotic fibrosarcoma oncogene homolog
MGUS: monoclonal gammopathy of undetermined significance
MM: Multiple myeloma
MMSET: Multiple myeloma SET domain
MTTP: Microsomal triglyceride transfer protein
NF-κB: Nuclear factor kappa-light-chain-enhancer of activated B cells
NGS: Next generation sequencing
PL: Phospholipid
PLTP: Phospholipid transfer protein
PPARγ: Peroxisome proliferator–activated receptor γ
PUFA: Polyunsaturated fatty acid
RB1: Retinoblastoma protein 1
SDF-1: Stromal cell-derived factor-1
SM: Smoldering or asymptomatic myeloma
SR-BI: Scavenger receptor class B type I
TC: Total cholesterol
TGFβ1: Transforming growth factor beta 1
TNF: Tumor necrosis factor
TP53: Tumor protein p53
TRLs: Triglyceride-rich lipoproteins
TGs: Triglycerides
VEGF: Vascular endothelial growth factor
VLDL: Very low density lipoprotein
VLDL-C: Very low density lipoprotein-cholesterol.
